# Successful Recovery from Meningoencephalitis Associated with Archetype-like JC Virus in a Lung Transplant Recipient: Case Report and Review of the Literature

**Published:** 2025-04-11

**Authors:** Julie C Gudenkauf, Elizabeth Wagstaff, Erik J. Arneson, Christine Gill, Aaron N. Gillman, Hillel Haim, C. Sabrina Tan

**Affiliations:** 1 Department of Neurology, University of Iowa, Iowa City, USA; 2 Department of Microbiology and Immunology, University of Iowa, Iowa City, USA; 3 Department of Internal Medicine, University of Iowa, Iowa City, USA

**Keywords:** JC virus, Transplant, Immunosuppression, Meningoencephalitis, Case report, Review

## Abstract

Meningoencephalitis due to JC polyomavirus (JCV) is rare and delays in diagnosis could lead to potentially fatal outcomes in immunosuppressed patients. We present a case of an HIV-negative lung transplant recipient who presented with neurological deficits, including aphasia and right-sided weakness. Brain imaging lacked demyelination usually diagnostic of progressive multifocal leukoencephalopathy (PML), the disease most often associated with JC virus, however cerebrospinal fluid (CSF) metagenomic analysis confirmed a high JC viral load, suggestive of JCV-associated meningoencephalitis. After reducing immunosuppression, the patient showed significant neurological improvement within three months and full recovery by 6 months. The JCV genome sequenced from patient’s plasma and CSF were identical and resembled the “nonpathogenic” archetype in the non-coding region but shared homology in the coding region with the classically-considered neurotropic strains detected in those with PML. These findings suggest that mutations in the virus’s noncoding region are not necessary for neuropathogenesis. We also review other cases of JCV-associated meningitis and encephalitis, which, in contrast to our case, were all fatal. Clinicians should consider JCV testing in immunosuppressed patients with encephalopathy and focal neurological deficits, even in the absence of significant brain radiographic abnormalities.

## Introduction

Human polyomavirus JC virus (JCV) is a highly prevalent DNA virus typically found in the archetypal form in human kidneys and occasionally excreted in the urine of immunocompetent hosts [1,2]. This archetypal form has historically been considered nonpathogenic; JCV variants, emerging with immunosuppression, contain changes in the noncoding control region (NCCR) that are thought to be required for viral infection of the central nervous system (CNS). Rearrangements in the NCCR are detected in JCV isolates from cerebrospinal fluid (CSF) and brain tissues from patients with progressive multifocal leukoencephalopathy (PML) and granule cell neuronopathy (GCN) [1,3,4]. Few reported cases of atypical CNS disease including JCV meningitis and encephalitis in which an archetype-like NCCR was present, all had rapid fatal outcomes [2,5–7].

We present a patient who developed JCV-associated meningoencephalitis 11 months after bilateral lung transplantation. Sequencing of the JCV isolated from her CSF and plasma revealed an architype-like NCCR, most similar to a sequence described in a case of JCV-associated nephropathy [8]. Unlike the reported fatal cases, our patient fully recovered within 6 months and did not have the classic presentation of brain demyelination.

### Case Presentation

We present a 61-year-old woman with a history of bilateral lung transplant for pulmonary hypertension, with additional past medical history of smoking and thyroidectomy. She had no other significant personal or family medical history, and had no history of HIV, alcohol consumption or recreational substance use. Her immediate post-transplantation course was without complication except for a perioperative Horner syndrome. She was adherent to her anti-rejection regimen (daily prednisone 7.5 mg and twice daily tacrolimus 4 mg/3 mg and mycophenolate 750 mg/500 mg) and to her prophylaxis regimen (daily valganciclovir, twice daily isavuconazole, daily atovaquone due to allergy to sulfa, and azithromycin three times weekly).

**Figure 1:**
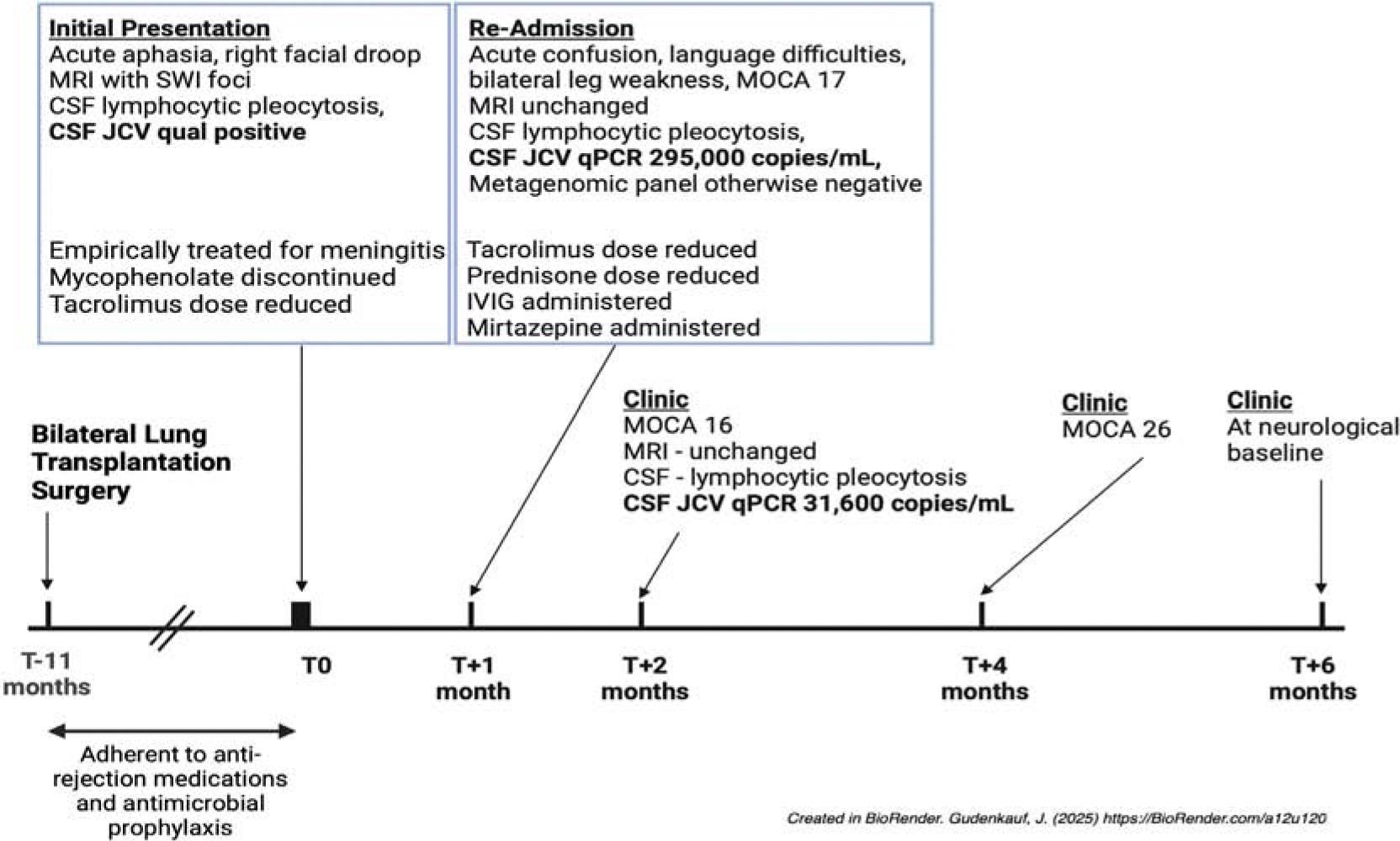
Timeline of our patient’s case.

**Figure 2:**
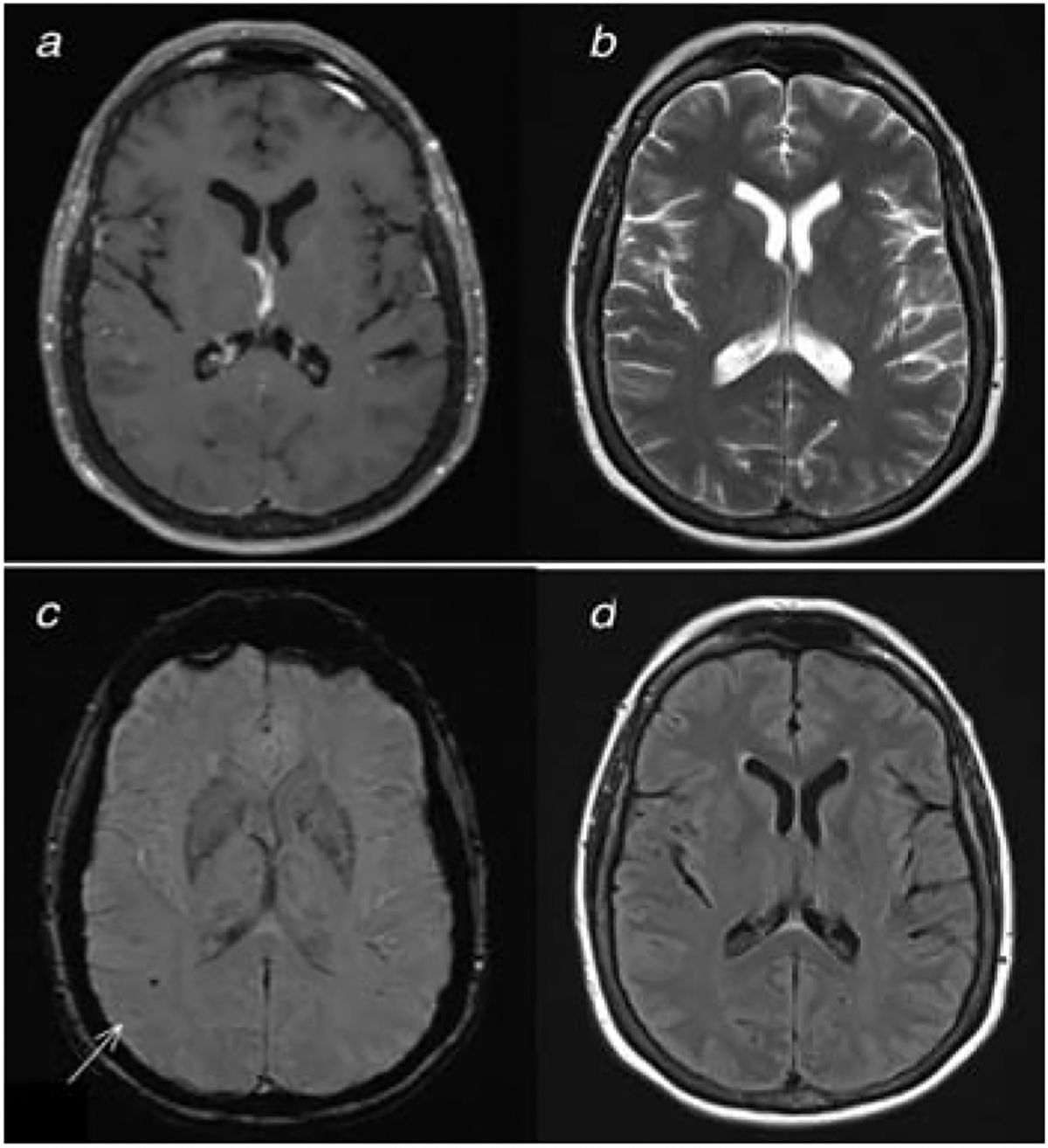
A) Brain MRI with T1-post contrast, B) T2, C) susceptibility weighted imaging (SWI), and D) T2-fluid-attenuated inversion recovery (FLAIR) was within normal limits, except for a SWI focus in the right temporal lobe, suggestive of microhemorrhage (C arrow).

Eleven months post-transplantation, she presented to a local emergency room with acute confusion, slurred speech, right facial droop, and right-hand paresthesia (Figure 1-timeline). Her initial exam was notable for global aphasia and a mild right facial droop. The severity of these deficits fluctated over time. Brain magnetic resonance imaging (MRI) demonstrated few scattered foci of susceptibility weighted imaging (SWI) signal suggestive of microhemorrhages in the left peri-Rolandic area, right temporal lobe, and right cerebellum (Figure 2 - MRI). She had no prior brain MRIs for comparison. Electroencephalogram (EEG) showed generalized slowing. Serum white blood cell count, basic metabolic panel and liver enzymes were normal. Her total serum IgG was low at 564 mg/dL (ref 700–1,600 mg/dL). She had normal TSH, Vitamins B1, B12, and D levels. CSF analysis upon arrival revealed 14 white blood cells (WBC/mm^3^; 90% lymphocytes), 1,000 red blood cells (RBC/mm^3^), protein 59 mg/dL, and glucose 68 mg/dL. CSF Meningitis/Encephalitis panel (BioFire Diagnostic, Inc.), cryptococcal antigen, bacterial and fungal cultures, and West Nile Virus (WNV) antibody were all negative. She was treated empirically for meningitis with antibiotics and acyclovir, which was narrowed to acyclovir monotherapy after the lumbar puncture. She started to improve after several days of treatment. Ultimately, CSF JCV qualitative polymerase chain reaction (PCR; Mayo Clinic) resulted positive. She was discharged home after discontinuation of mycophenolate as well as reduction in her tacrolimus dose.

**Figure 3:**
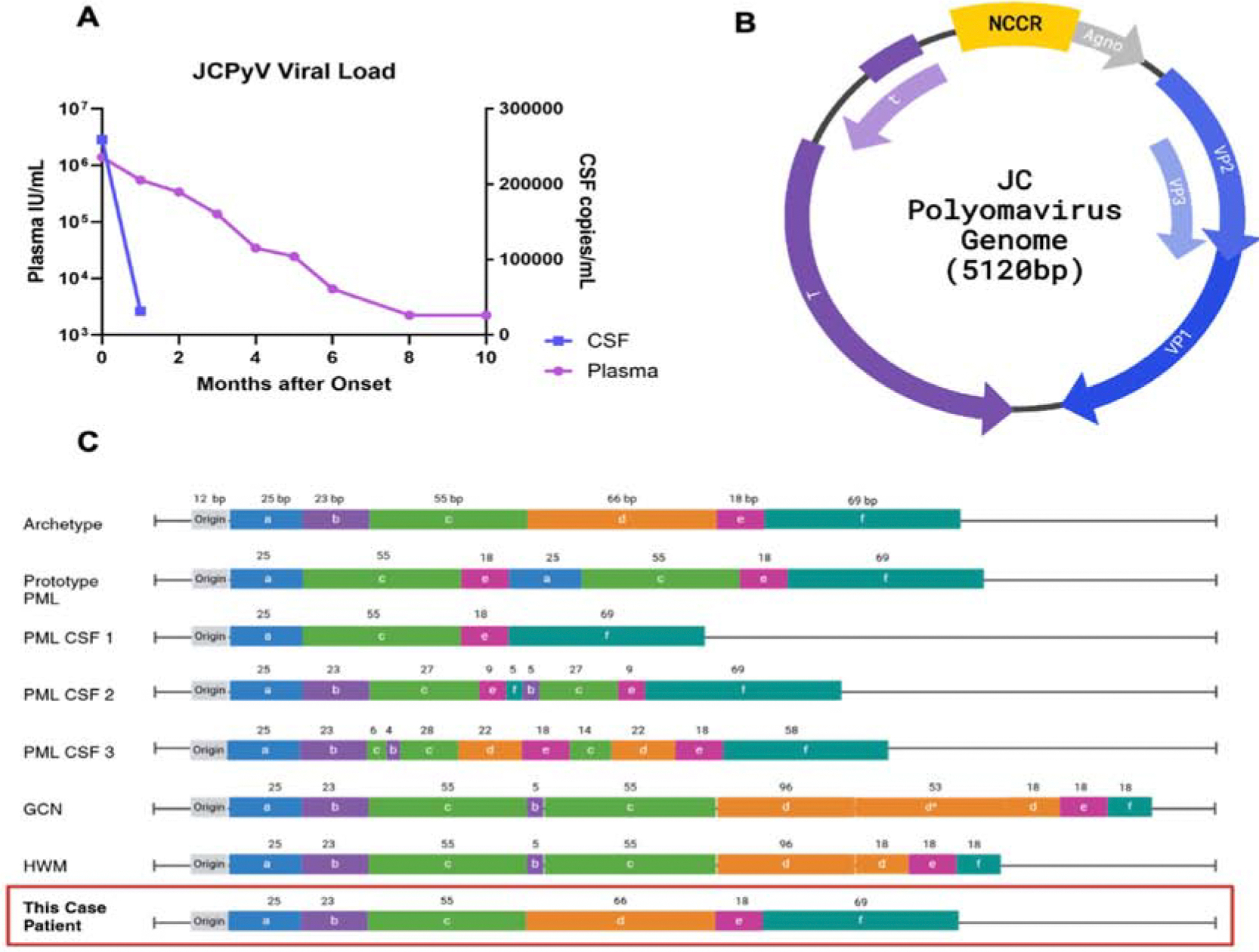
A) Quantification of JCPyV by PCR showed slow decrease in blood and a more rapid decrease in the CSF with reduction in immunosuppression. CSF was not tested after 1 month. B) A simplified map of the JCPyV genome showing the location of the noncoding control region (NCCR) in relation to protein coding regions. C) Diagram comparing various rearrangements in the NCCR region. The NCCR is divided into 6 sequence sections labeled a-f according to previous classifications [16,17]. The numbers above the segments describe the base pair length. JCPyV isolated from our patient (JCME) had Archetype NCCR. The archetype sequence [17] shows the length and location of these sections without rearrangement. Prototype PML shows the rearrangement of MAD-1 (the first JCPyV sequence published) [15,17]. Three common NCCR rearrangements from PML positive CSF show some of the variety of rearrangements seen in patient isolates [17]. Granule cell neuronopathy (GCN) and hemorrhagic white matter (HWM) show the rearrangements of two isolates from one patient with GCN. GCN shows the sequence isolated from infected granule cell neurons and HWM shows the sequence found in hemispheric white matter [3]. The asterisk (*) represents an insertion with many base pair changes from the archetype sequence. Our case, at the bottom of the panel, shows the NCCR arrangement we identified. PML isolates often contain deletions in the b and/or d segments as well as partial or complete insertions of repeated regions. GCN and HWN both contain large insertions. The NCCR sequence in our case contains no insertions or deletions.

Ten days after discharge, the patient returned with severe vomiting, confusion, language difficulties, bilateral leg weakness, ataxia, and subjective tongue heaviness. Repeat brain MRI was unchanged. CSF analysis revealed 24 WBC/mm^3^ (96% lymphocytes), <500 RBC/mm^3^, protein 85 mg/dL, and glucose 59 mg/dL. CSF ME panel was negative. CSF JCV quantitative PCR (qPCR) revealed 295,000 copies/mL (Eurofins-Viracor) (Figure 3 - virus). A CSF metagenomic panel was sent to the University of California San Francisco, which only detected “human polyomavirus 2 (JC polyomavirus)” DNA. Nucleic acids from RNA viruses, bacteria, fungi, and parasites were not detected. Given clinical concern for JCV-associated meningoencephalitis, her tacrolimus dose was further reduced as was her daily prednisone dose. Intravenous immunoglobulin (IVIG) was administered given her hypogammaglobulinemia. Mirtazapine was also given based on in vitro evidence that JCV uses the serotonin 5HT2 receptor for entry [9,10]. Prior to discharge she scored 17/30 on the Montreal Cognitive Assessment (MOCA), with deficits in visuospatial and executive function, as well as memory.

At her clinic follow up appointment two weeks after discharge, she was still frequently forgetful, had trouble with word-finding, intermittent confusion, and tremors that fluctuated in severity and were most noticeable when she was using her hands. At this appointment, her brain MRI was unchanged and CSF showed 41 WBC/mm^3^ (80% lymphocytes), <500 RBC/mm^3^, protein 62 mg/dL, glucose 52 mg/dL. CSF JCV qPCR of 31,600 copies/mL. Repeat MOCA was similar, with a score of 16/30.

Four months after initial presentation, her MOCA improved to 26/30. Her plasma JC virus slowly trended downwards over 6 months from 1,391,409 IU/mL to 6,551 IU/mL (Figure 3- virus). At her six-month appointment, she felt completely back to her baseline. She declined further CSF assessments.

### JCV Genomic Sequencing (Plasma and CSF)

We analyzed the JCV sequences amplified from the patient’s CSF and plasma (Supplemental Methods). JCV was not detected in her urine. Multiple sequence alignments were performed to compare our assembled genomes to archetype JCV, as well as isolates associated with neuropathogenesis (Figure 3).

JCV genomes amplified from the patient’s CSF and plasma revealed complete nucleotide homology to each other (GenBank PQ137416, PQ085639) (Figure 3). The NCCR region had an archetype-like profile, without the insertion and deletion patterns typical of the neurotropic JCV isolates. Only a single nucleotide substitution was observed in the patient’s NCCR relative to the JCV archetype strain, (G226A). However, in the coding region of the JCV genome, our patient’s strain diverged considerably from the JCV archetype (105 substitutions); but had closer homology with the MAD1 neuropathogenic strain (23 substitutions).

## Discussion

We present a complex and unique case of a clinical meningoencephalitis with high quantities of archetype-like JCV virus in the CSF of an immunocompromised post-transplantation patient. Our patient had an acute onset of focal neurological symptoms eleven months after transplantation, brain MRI without T2-FLAIR hyperintensities, contrast enhancement, or cerebellar abnormalities, and a CSF lymphocytic pleocytosis. She had slight symptomatic improvement with a course of empiric meningitis antimicrobials and adjustments in her anti-rejection regimen, however she quickly worsened with frank meningitis symptoms and had an elevated CSF JCV viral load >250,000 copies/mL. Metagenomic sequencing of the CSF confirmed high quantities of JCV viral DNA and was negative for any other viral, bacterial, fungal, or parasitic genetic material. Her clinical course improved after further reduction of her anti-rejection regimen and trials of IVIG and mirtazapine. The patient’s clinical symptoms and CSF findings were most consistent with JCV-associated meningoencephalitis.

JCV is known to infect oligodendrocytes and astrocytes in PML [1] and cerebellar granule cells in GCN [3,4], however, more recently, JCV is being recognized as infecting cerebral cortical neurons in JCV encephalitis and the leptomeninges in JCV meningitis. There are a number of cases with histopathological evidence of JCV isolation in the cortex and meninges [2,5–7,11]. Of note, patients in these cases had high quantities of JCV DNA the CSF; much higher than levels seen in PML [7]. There are also cases of clinically undifferentiated meningoencephalitis in which patients had very high quantities of JCV DNA in the CSF [12–14]; these cases lack histopathological confirmation, but it is presumed that the brain parenchyma and meninges were infected with JCV. Similarly, while we did not have histopathological confirmation of JCV isolation in the cortex or meninges, our patient’s neurological manifestations and her high CSF levels of JCV, in the absence of any other microorganism identified by metagenomic testing, are highly suggest JCV meningoencephalitis.

Our patient had unique imaging findings that have not previously been described in association with JCV CNS infections. MRI findings classically associated with JCV infections of the CNS are T2/FLAIR white matter hyperintensities in PML and cerebellar atrophy in GCN [1,3,4]. In JCV-associated meningitis or encephalitis, there may be gray matter lesions [2], hydrocephalus and sulcal FLAIR hyperintensities [7], or imaging may be normal [6]. Our patient had lobar and cerebellar microhemorrhages seen on SWI. It is unclear if these were present prior to JCV infection and were thus incidental, or if they truly represent lesions related to the JCV infection, given the lack of pre-morbid brain images for comparison.

An exceptional feature of this case is the genomic profile of the virus isolated from the CSF. Mutations in the NCCR of JCV have been associated with neurotropism; JCV isolates from brain, including MAD1, HWM and GCN1 all have multiple insertions and deletions in the NCCR region relative to the virus usually isolated from urine [3,15]. In our patient, the JCV genomes from CSF and plasma were identical, and also nearly identical to the archetype strain, but within the coding region of the genome, significant homology was observed with the PML-associated MAD1 strain. Our patient’s JCV genome was most similar to a sequence described in a case of JCV-associated nephropathy [8]. The homology with the NCCR of the archetype virus, and the coding region with MAD1 suggest that neuropathogenic properties of our strain may be due to the changes in the coding region. Perhaps destruction of oligodendrocytes and astrocytes may in part depend on NCCR rearrangement. JC viral replication depends on host transcription factors binding the NCCR region. Thus, changes in this region could direct organ-specific pathogenesis.

What is perhaps the most impressive feature of this case is our patient’s clinical recovery. JCV CNS infections are associated with high mortality, especially JCV encephalitis, meningitis, and meningoencephalitis which are uniformly fatal in reported cases (Table 1) [2,5–7]. Our patient had complete neurological recovery by 6 months after the identified infection. Perhaps the early diagnosis of JCV infection and prompt reduction in immunosuppressants along with IVIG infusion and mirtazapine led to sufficient recovery of her immune system to contain the virus. Future studies to establish prognostic factors in JCV-related CNS disease are warranted.

There are a number of limitations to this case study. First, we do not have biopsy tissue confirming JCV isolation in the cerebral cortex or the meninges. Second, metagenomic sequencing in the CSF was sent after she had completed a course of empiric acyclovir for possible viral meningitis. It is possible that there was another infectious agent initially involved that was treated by the meningitis antimicrobials, such as HSV, however this is unlikely given the patient’s continued neurological deterioration and absence of HSV-associated imaging findings on her brain MRI. Lastly, this is a single case report. Larger studies involving more patients with JCV infections and greater knowledge of both NCCR and coding region mutations in all JCV diseases will be important to understand the spectrum of JCV associated CNS disorders and viral and host factors contributing to the neuropathognesis.

## Conclusion

We report a case of JCV-associated meningoencephalitis without typical brain MRI features in an immunosuppressed patient who demonstrated complete neurological recovery and, importantly, the JCV strain had archetype-like NCCR but with neuropathogenic mutations in the coding region. The findings suggest that NCCR mutations are not required for JCV neuropathogenesis, and that elements in the JCV coding region may also contribute to the neuropathogenesis of this virus. JCV infection should be considered in immunosuppressed patients presenting with neurological deficits, even with normal brain images. Future research is critically needed to elucidate the prevalence of JCV DNA in the CSF of patients with encephalitis or aseptic meningitis.

## Supplementary Material

Supplemental Information

## Figures and Tables

**Table 1:** Cases of JCV-associated encephalitis, meningitis, and meningoencephalitis confirmed by histopathology, and the associated JCV sequences. All cases were HIV-negative.

Reference	Wüthrich et al., 2009; Dang et al., 2012b	Reoma et al., 2019	Agnihotri et al., 2014	Present Case
Patient	74-year-old woman with non-small cell lung cancer who completed chemotherapy 5 months before her neurological symptoms of aphasia and progressive cognitive dysfunction.	16-year-old girl with refractory aplastic anemia complicated by EBV reactivation requiring rituximab, along with corticosteroids and ruxolitinib for late onset acute skin graft-versus-host disease. Two months later she had a seizure-like episode. EEG with no seizures.	67-year-old woman with a history of recurrent infections had 2 months of progressively worsening headache, vomiting, urinary incontinence, and weakness in her legs. She became increasingly forgetful, could not recognize her family members, and increasingly lethargic.	61-year-old woman who presented with neurological complications eleven months after lung transplant. Upon reduction of her anti-rejection regimen, she had near complete neurological recovery.
Outcome	Death	Death	Death	Recovery
MRI Brain	Lesions restricted to hemispheric gray matter	No obvious abnormalities	Abnormal signal in subarachnoid space within the sulci of the cerebral hemispheres; hydrocephalus	No obvious abnormalities
CSF	2 WBC, 0 RBC, Protein 47.8 mg/dL, Glucose 67 mg/dL. Qualitative PCR confirmed the presence of JCV.	1 WBC and protein 98 mg/dL. JCV PCR 445,490 copies/mL.	10 WBC, 244 RBC, Protein 61 mg/dL, Glucose 50 mg/dL. JCV PCR 8.9 × 10^6^ copies/mL.	14 WBC, 1,000 RBC, Protein 59 mg/dL, and Glucose 68 mg/dL. Qualitative PCR confirmed the presence of JCV.
Brain Autopsy	Diffuse cortical involvement of both cerebral hemispheres, numerous JCV-infected astrocytes at the gray–white junction and in the cortex, productive JCV infection in cortical pyramidal neurons.	Positive VP1 staining in hippocampal and cortical neurons as well as in cortical glial and endothelial cells. VP1 was also detected by Western blot analysis in brain samples dissected from the occipital and frontal lobes. VP1 levels were higher in gray than in white matter. JCV load per milliliter of CNS autopsy tissue surpassed 1 billion copies in all sampled areas.	Productive JCV infection of leptomeningeal and choroid plexus cells, and limited parenchymal involvement.	Not performed
JCV Sequence	Unique 143 bp deletion in the JCV Agno gene, JCV_CPN1_ (Reported in Dang et al 2012).	Novel 12 bp insertion in a noncoding region in the intergenic area between VP1 and large T antigen of the early mRNA transcript. Within the noncoding early mRNA, there was a gain of 18 transcription binding factor sites in this unique viral sequence, 50% of which were found within or overlapping the 12bp inserted region.	Archetype-like NCCR bearing a small deletion in the 66 bp insert in 9 of 10 clones. A minority NCCR (1 of 10 clones) with deletion of nt 37–209 was also observed. A total of 4 silent mutations were found in the VP1 capsid gene C-terminus.	Archetype-like NCCR with one point mutation at nucleotide 217. Various point mutations seen across VP1 and T Ag similar to MAD-1 however few mutations resulted in amino acid changes. Most similar to a case of nephropathy (Genbank MF662195).
