# Supplementary material for: Successful Recovery from Meningoencephalitis Associated with Archetype-like JC Virus in a Lung Transplant Recipient: Case Report and Review of the Literature
Source: Ann Clin Case Rep. Author manuscript; Available in PMC 2026 May 9. (PMC13155386)
Supplement: Supplemental Information [file NIHMS2144080-supplement-Supplemental_Information.pdf]

Supporting Information

Successful Recovery from Meningoencephalitis Associated with Archetype-like JC Virus in a Lung Transplant Recipient: Case Report and Review of the Literature

Julie C. Gudenkauf, Elizabeth Wagstaff, Erik J. Arneson, Christine Gill, Aaron N. Gillman, Hillel Haim and C. Sabrina Tan

Published in: Annals of Clinical Case Reports. 2025;10(1):2736.
DOI: 10.25107/2474-1655.2736

Supplemental Methods

PCR amplification of the JCV genome

JCV DNA was purified from cerebrospinal fluid (CSF) and plasma samples using the QIAprep Spin Miniprep kit (Qiagen). Different regions of the JCV genome were amplified using the primer pairs shown in the table below. Primers were designed to produce fragments based on the MAD1 reference genome (Accession number J02226). All PCR reactions were performed using PrimeSTAR Max DNA Polymerase Master Mix (Takara, R045) in a 50 µl reaction volume containing 0.3 µM of each primer and 1 µl of extracted template from plasma or CSF. Cycling conditions included 30 cycles with an extension time of 10 sec/kb. For the nested PCR, we used 1 µl of the first-round PCR product as template. Annealing was performed at the temperatures indicated below for 5 sec.

Primer Pair	First Primer Sequence (5'to 3')	Second Primer Sequence (5'to 3')	Annealing Temp.	Nucleotide Position (MAD1)
1	CAGATCTACAGGAAAGTCTTTAGGGTC	CTTCTATAGTAGCAGCAGCCTCTC	56.9° C	4241 to 643
2	CACAGGTGAAGACAGTGTAGACG	GCCACTAATCCTTCAGTGCATTG	56.9° C	396 to 4710
3	CACAGGTGAAGACAGTGTAGACG	GACCCTAAAGACTTTCCTGTAGATCTG	56.9° C	396 to 4267
4	CAATGCACTGAAGGATTAGTGGC	CGTCTACACTGTCTTCACCTGTG	57.1° C	4688 to 418
5	CCTCACACTTGGTTTCCAAGGCATACTGTGTAACTAATTTCC	CTATATCCCAACTGAGCAATAGCACTACCACCAGTTACAG	65.3° C	3817 to 824
6	GCTGACACTCTATGTCTATGTGGTGTTAAG	CTACAGTAGCAAGGGATGCAATTTCAACTTC	59.9° C	4047 to 670
